# Temporal changes in gait parameters and muscle activation during sustained walking in children with spastic cerebral palsy

**DOI:** 10.3389/fped.2026.1865485

**Published:** 2026-09-09

**Authors:** Lina Zhang, Li Yu, Haibo Guo, Pei Ma, Fang Liu, Axel Kalpen, Wei Li

**Affiliations:** 1 Department of Rehabilitation Medicine, Shandong Medical and Pharmaceutical University Hospital, Binzhou Shandong Province, China; 2 Department of Child Health and Developmental Behavior, Shandong Medical and Pharmaceutical University Hospital, Binzhou Shandong Province, China; 3 Novel GmbH, Munich, Germany

**Keywords:** cerebral palsy, gait analysis, gait variability, motor fatigue, surface electromyography

## Abstract

**Objective:**

Temporal changes in spatiotemporal gait parameters and muscle activation may reflect progressive gait deterioration during prolonged walking in children with spastic cerebral palsy.

**Methods:**

A portable gait analysis system and surface electromyography were used to evaluate gait characteristics during a 3-minute walking protocol in 15 children with SCP and 15 age-matched healthy controls. Participants walked at a self-selected speed on a level surface. Spatiotemporal gait parameters, energy expenditure, and bilateral gastrocnemius and tibialis anterior muscle activation were assessed at predefined time points. Linear mixed-effects models were applied to examine group-by-time interactions while accounting for repeated measurements within participants.

**Results:**

Linear mixed-effects model analysis revealed significant Group × Time interactions for stance phase, swing phase, step length, lower-limb forward acceleration, foot clearance angle, and walking velocity (all *P* < 0.05), indicating divergent temporal trajectories between children with SCP and healthy controls. No significant Group × Time interactions were observed for single-step time, gait cycle duration, or cadence (all *P* > 0.05). For energy expenditure, a significant Group × Time interaction (*P* = 0.047) indicated that the SCP group maintained elevated metabolic cost into the third minute while controls exhibited a declining trajectory. Similarly, significant Group × Time interactions were observed for bilateral gastrocnemius (*P* = 0.002) and tibialis anterior (*P* = 0.006) normalized root mean square amplitudes, reflecting a greater temporal decline in muscle activation in the SCP group from the second minute onward.

**Conclusion:**

Children with SCP may exhibit gradual changes in spatial-temporal gait parameters and muscle activation patterns during the later stages of a long walking task. These findings suggest that selected gait and EMG parameters in children with SCP begin to change after approximately 2 min of continuous walking, indicating a potential time window for closer monitoring or structured rest breaks during rehabilitation. Further studies incorporating direct fall-risk assessment and validated fatigue measures are warranted before definitive clinical recommendations can be made.

## Highlights

1. Temporal gait changes emerge after 2 min in SCP
2. Step time and stance phase increase during prolonged walking
3. Bilateral ankle EMG amplitude declines from 2 min
4. Foot clearance increases with walking duration
5. Energy cost remains elevated in the third minute

## 1 Introduction

Cerebral Palsy (CP) comprises a group of persistent central motor and postural development disorders resulting from non-progressive injury to the developing fetal or infant brain. CP is categorized into spastic, dyskinetic, ataxic, and mixed subtypes (1). Spastic cerebral palsy (SCP) accounts for more than half of all cases and is the primary focus of clinical management and research (2).

SCP is a lifelong motor disability characterized by abnormal muscle tone, reduced muscle strength, secondary contractures, and musculoskeletal deformities (3). These impairments may contribute to reduced walking efficiency, increased fatigability, and a higher risk of falls in affected children (4). Maintaining stable and efficient walking patterns is therefore essential for safe ambulation. Previous studies have demonstrated that gait variability, typically defined as stride-to-stride fluctuations in spatiotemporal gait characteristics, is associated with gait stability and functional mobility in individuals with neurological disorders, including children with cerebral palsy (5). Gait variability reflects fluctuations in spatiotemporal gait characteristics between consecutive cycles and comprises two distinct components: deterministic variations arising from systematic adjustments in motor commands, and stochastic variations attributable to neuromuscular noise inherent in the sensorimotor system (6). In typically developing children, supraspinal control mechanisms effectively suppress stochastic noise to maintain consistent gait patterns. In children with SCP, however, impaired central drive and altered motor-unit recruitment reduce the capacity to attenuate this noise, resulting in a greater stochastic contribution to gait variability. This inability to suppress neuromuscular noise is considered a central pathophysiological mechanism underlying the increased gait variability observed in SCP (7).

Previous studies have consistently demonstrated that children with CP exhibit signs of lower-limb muscle fatigue, altered neuromuscular activation, and spatiotemporal gait deterioration during prolonged walking (8, 9). For example, Eken et al. (10) observed accelerated EMG median-frequency declines in the lower leg during a 5-minute overground walk, and Ettema et al. (9) reported progressive EMG changes and increased muscle fatigue during a 6-minute treadmill protocol in this population. Similarly, fatigue-related gait adaptations—including prolonged stance phase and reduced swing phase—have been documented following sustained walking exercises (11). Nevertheless, the precise minute-by-minute temporal onset at which these gait and EMG parameters begin to diverge from typical patterns during continuous overground walking remains poorly characterized. Identifying this temporal threshold may provide important insights into the mechanisms underlying walking fatigue and help optimize rehabilitation strategies.

This study was designed to determine the temporal onset of gait deterioration in children with SCP, thereby establishing evidence-based walking–resting intervals for daily activities and rehabilitation, and to provide a theoretical and experimental foundation for clinical practice.

## 2 Materials and methods

### 2.1 Participants

Participants were divided into an SCP group and a control group. The SCP group included 15 children with SCP recruited from the outpatient clinic of the Children's Rehabilitation Center, Affiliated Hospital of Binzhou Medical College. All children with SCP included in this study had spastic diplegia, with bilateral lower-limb involvement. Although bilateral gait parameters were assessed, clinical lateralization, including dominant limb or asymmetry based on standardized clinical evaluation, was not specifically determined. All children with SCP had spastic diplegia with bilateral lower-limb involvement. Clinical lateralization was not formally determined. To ensure baseline symmetry, two experienced pediatric rehabilitation physicians independently assessed each child using the Edinburgh Visual Gait Score (EVGS) (12), with emphasis on limb-symmetry items. Only children with an EVGS limb-symmetry subscore ≤ 2 and a quantitative step-length symmetry index (|left − right|/mean × 100%) < 15% during the initial 10-second walking period were retained. We acknowledge that spastic diplegia may mask underlying asymmetrical cortical involvement; nevertheless, this screening was implemented to minimize gross kinematic asymmetry that could confound bilateral comparisons. The control group comprised 15 healthy children recruited during the same period, matched by sex, age, and body mass index (BMI).

Inclusion criteria for the SCP group were: (1) diagnosis of CP based on the Chinese CP Rehabilitation Guidelines (2022); (2) Gross Motor Function Classification System (GMFCS) level I–II; (3) ability to follow instructions and cooperate; (4) age 3–6 years; and (5) informed consent provided by a legal guardian. Exclusion criteria were: (1) inability to cooperate with assessments; (2) visual or hearing impairment; (3) severe epilepsy or other major comorbidities affecting rehabilitation; (4) use of anticonvulsants or botulinum toxin within the preceding 6 months; or (5) inability to undergo gait analysis. Controls had normal cognition, no musculoskeletal disorders, and no history of trauma.

The study protocol was approved by the ethics committee (approval number: KT-58). Written informed consent was obtained from all participants' guardians.

Baseline characteristics are presented in Table 1. Baseline demographic, anthropometric, and clinical characteristics were comprehensively documented. For the SCP cohort, this included Gross Motor Function Classification System (GMFCS) level, CP subtype, spasticity severity as graded by the Modified Ashworth Scale (MAS) for the ankle plantarflexors and hip adductors, use of ankle–foot orthoses (AFO) and assistive devices, history of physical/occupational therapy (PT/OT) within the preceding 6 months, history of orthopedic surgery, and history of botulinum toxin A (BoNT-A) injection (with the last injection administered ≥6 months prior to enrolment). Demographic and musculoskeletal parameters comprising sex, age, height, weight, body mass index (BMI), manual muscle strength [Medical Research Council (MRC) scale, 0–5], hip abduction range of motion (ROM), and knee extension ROM were assessed in both groups. No significant between-group differences were observed in age, height, weight, BMI, muscle strength, hip abduction ROM, or knee extension ROM (all *P* > 0.05), indicating that the two cohorts were comparable with respect to these baseline characteristics.

**Table 1:** Baseline characteristics.

Characteristic	Control (*n* = 15)	SCP group (*n* = 15)	*P*
Gender (*n*, %)			0.690
Male	10 (66.7)	11 (73.3)	
Female	5 (33.3)	4 (26.7)	
Age (years)	5.07 ± 0.88	4.67 ± 1.23	0.317
Height (m)	1.10 ± 0.14	1.13 ± 0.13	0.511
Weight (kg)	20.36 ± 2.98	22.67 ± 6.87	0.306
BMI (kg/m^2^)	17.68 ± 3.22	17.55 ± 2.63	0.910
GMFCS Grading			
Ⅰ	／	5 (33.33)	／
Ⅱ	／	6 (40.00)	／
Ⅲ	／	4 (26.67)	／
MAS			
Ankle plantarflexors	／	1.82 ± 0.44	／
Hip adductors	／	1.37 ± 0.63	／
Use of AFO, *n* (%)	／	3 (20.00)	／
Use of assistive devices, *n* (%)	／	1 (6.67)	／
PT/OT within 6 months, *n* (%)	／	12 (80.0)	／
History of orthopedic surgery, *n* (%)	／	1 (6.7)	／
History of BoNT-A, *n* (%)*	／	3 (20.0)	／
Muscle strength (MRC, 0–5)	4.66 ± 0.22	4.61 ± 0.25	0.563
Hip abduction ROM (°)	43.33 ± 3.72	42.73 ± 3.92	0.666
Knee extension ROM (°)	5.71 ± 1.45	5.4 ± 0.98	0.571

* Last injection ≥6 months prior to enrolment.

### 2.2 Sample size justification

This study was designed as an exploratory observational investigation aiming to characterize the temporal dynamics of gait parameters and neuromuscular activation during continuous walking in children with SCP, rather than to test a prespecified hypothesis; therefore, no *a priori* sample size calculation was performed. The sample size was determined according to established recommendations for pilot and feasibility studies, which suggest a minimum of 12 participants per group when reliable prior effect-size estimates are unavailable (13, 14), and was further informed by comparable gait and surface EMG studies in children with cerebral palsy, which typically enrolled 10–15 participants per group owing to the difficulty of recruiting homogeneous pediatric populations and the complexity of biomechanical assessments involving repeated measurements (10, 11).

The repeated-measures design, in which each participant completed six trials across four time points with bilateral measurements, substantially increased the information contributed by each participant, and linear mixed-effects models were used to account for within-subject correlations. Sensitivity analysis indicated that 15 participants per group provided 80% power to detect a standardized between-group effect of Cohen's d = 1.06 at a two-sided *α* of 0.05; the observed effect sizes for the principal gait and energy-expenditure outcomes ranged from d = 1.25–2.36, exceeding this threshold. Although smaller effects cannot be excluded, particularly for secondary outcomes, the findings should be interpreted as hypothesis-generating and require confirmation in larger, adequately powered multicenter cohorts.

### 2.3 Data collection

Gait parameters were assessed using a portable intelligent gait analysis system (IDEEA, Minisun, USA), consisting of five sensors connected to a central recorder. Sensors were affixed to the sternum, anterior thighs, and feet with medical tape, and linked to the waistband-mounted recorder via flexible cables. Participants walked back and forth on a 20 m × 2 m firm mat placed on a hard, even laboratory floor at their self-selected speed for three minutes. To isolate steady-state straight-line walking, the first and last two steps of each 20-m straight segment were discarded to eliminate acceleration and deceleration transients. Turning phases (approximately 3–4 steps at each end) were identified using the IDEEA system's gyroscope-derived directional-change detection and were manually verified by two independent researchers against video recordings. Only straight-line steady-state gait cycles were retained for spatiotemporal parameter calculation. EMG segments corresponding to turning phases were likewise excluded from the RMS analysis. Before testing, all participants rested in a seated position for at least 10 min after arrival to avoid pretest fatigue.

Each participant completed six 3-minute walking trials, yielding a total walking exposure of 18 min per session, with approximately two minutes of seated rest between consecutive trials. We acknowledge that a 2-minute seated rest is physiologically insufficient for complete clearance of accumulated peripheral neuromuscular fatigue or restoration of intramuscular pH, particularly in children with SCP who exhibit reduced walking endurance. Consequently, Trial 6 likely began with greater residual fatigue than Trial 1. To mitigate this, the descriptive statistics in Tables 2–4 represent the within-participant mean across the six trials at each time point. The primary inferential analyses (Tables 5–7) were performed using linear mixed-effects models on the full dataset (all six trials), with Participant as a random intercept to account for within-subject correlations across trials and time points. We additionally tested a model that included Trial number (1–6) as a fixed effect to capture order-related fatigue accumulation; however, this term did not significantly improve model fit (likelihood ratio test, *P* > 0.05) and was omitted from the final parsimonious model.

**Table 2:** Comparison of gait parameters between the SCP and control groups.

Parameter	Left					Right				
	Control (*n* = 15)	SCP (*n* = 15)	*t*	*P*	Cohen's d (95% CI)	Control (*n* = 15)	SCP (*n* = 15)	*t*	*P*	Cohen's d (95% CI)
Single step time (seconds)										
10 s after start	0.50 ± 0.13	0.57 ± 0.09	1.715	0.098	0.564 (−0.167–1.294)	0.51 ± 0.14	0.57 ± 0.11	1.305	0.201	0.456 (−0.269–1.182)
1st minute	0.48 ± 0.09	0.49 ± 0.09^a^	0.304	0.763	0.105 (−0.612–0.821)	0.50 ± 0.10	0.51 ± 0.08	0.305	0.765	0.103 (−0.613–0.820)
2nd minute	0.46 ± 0.07	0.49 ± 0.08^a^	1.093	0.284	0.375 (−0.347–1.098)	0.45 ± 0.06	0.55 ± 0.07	4.201	<0.001	1.561 (0.737–2.385)
3rd minute	0.44 ± 0.05	0.51 ± 0.09	2.633	0.014	0.971 (0.212–1.731)	0.45 ± 0.05	0.56 ± 0.09	4.138	<0.001	1.477 (0.663–2.290)
*F*	1.235	2.801				1.723	1.317			
*P*	0.306	0.048				0.173	0.278			
Stance phase (%)										
10 s after start	61.81 ± 4.18	61.42 ± 5.11	0.229	0.821	−0.083 (−0.799–0.633)	62.91 ± 5.69	62.52 ± 7.46	−0.160	0.874	−0.058 (−0.774–0.657)
1st minute	60.33 ± 3.05	61.71 ± 4.42	0.995	0.328	0.366 (−0.356–1.088)	62.23 ± 5.03	63.01 ± 3.93	0.475	0.638	0.173 (−0.544–0.891)
2nd minute	58.15 ± 3.36^a^	62.17 ± 5.08	2.555	0.016	0.933 (0.177–1.689)	60.58 ± 2.97	66.43 ± 4.29^a^^,^^b^	4.341	<0.001	1.585 (0.758–2.413)
3rd minute	58.63 ± 3.76^a^	63.38 ± 4.47	3.148	0.004	1.150 (0.373–1.926)	60.54 ± 2.50	68.09 ± 4.15^a^^,^^b^	6.035	<0.001	2.204 (1.284–3.123)
*F*	3.216	0.490				1.174	4.067			
*P*	0.030	0.691				0.328	0.011			
Swing phase (%)										
10 s after start	38.19 ± 4.18	38.58 ± 5.11	0.227	0.822	0.083 (−0.633–0.799)	37.09 ± 5.69	37.48 ± 7.46	0.160	0.874	0.058 (−0.657–0.774)
1st minute	39.67 ± 3.05	38.29 ± 4.42	−1.001	0.325	−0.366 (−1.088–0.356)	37.77 ± 5.03	36.99 ± 3.93	−0.475	0.638	−0.173 (−0.891–0.544)
2nd minute	41.85 ± 3.36^a^	37.83 ± 5.08	−2.555	0.016	−0.933 (−1.689–−0.177)	39.42 ± 2.97	33.57 ± 4.29^a^^,^^b^	−4.341	<0.001	−1.585 (−2.413–−0.758)
3rd minute	41.37 ± 3.76^a^	36.62 ± 4.47	−3.148	0.004	−1.150 (−1.926–−0.373)	39.46 ± 2.50	31.91 ± 4.15^a^^,^^b^	−6.035	<0.001	−2.204 (−3.123–−1.284)
*F*	3.216	0.490				1.174	4.067			
*P*	0.030	0.691				0.328	0.011			
Gait cycle (s)										
10 s after start	1.06 ± 0.25	1.10 ± 0.13	0.505	0.618	0.184 (−0.533–0.902)	1.05 ± 0.27	1.12 ± 0.19	0.772	0.447	0.282 (−0.438–1.001)
1st minute	1.02 ± 0.18	1.04 ± 0.14	0.252	0.803	0.092 (−0.624–0.808)	1.00 ± 0.14	1.01 ± 0.11	0.377	0.709	0.138 (−0.579–0.854)
2nd minute	0.92 ± 0.15	1.04 ± 0.11	2.449	0.021	0.894 (0.141–1.647)	0.91 ± 0.13	1.02 ± 0.08	2.768	0.01	1.011 (0.248–1.774)
3rd minute	0.90 ± 0.11	1.04 ± 0.13	3.054	0.005	1.115 (0.342–1.888)	0.90 ± 0.11	1.06 ± 0.14	3.397	0.002	1.240 (0.454–2.026)
*F*	3.216	0.824				2.854	2.015			
*P*	0.030	0.486				0.062	0.122			
Step length (step length/leg length)										
10 s after start	0.91 ± 0.15	0.85 ± 0.07	0.321	0.769	0.111 (−0.616–0.837)	0.92 ± 0.15	0.85 ± 0.06	1.678	0.105	0.293 (−0.433–1.012)
1st minute	0.89 ± 0.10	0.81 ± 0.07	1.540	0.136	0.562 (−0.173–1.293)	0.88 ± 0.06	0.81 ± 0.07	2.941	<0.001	0.554 (−0.184–1.283
2nd minute	0.90 ± 0.10	0.78 ± 0.06	3.210	0.003	1.173 (0.391–1.952)	0.90 ± 0.08	0.78 ± 0.07	4.372	<0.001	1.441 (0.638–2.254)
3rd minute	0.90 ± 0.10	0.74 ± 0.07^a^^,^^b^	4.723	<0.001	1.72 4 (0.88–2.574)	0.88 ± 0.08	0.74 ± 0.06^a^^,^^b^	5.422	<0.001	0.512(−0.193–2.894)
*F*	0.076	7.104				0.567	7.647			
*P*	0.973	<0.001				0.640	<0.001			
Cadence (steps/minute)										
10 s after start	122.22 ± 5.00	123.75 ± 8.75	−1.059	0.299	−0.387 (−1.109–0.336)	122.87 ± 10.39	122.72 ± 7.96	−0.644	0.525	−0.235 (−0.953–0.483)
1st minute	124.50 ± 5.81	125.72 ± 11.02	−1.351	0.188	−0.493 (−1.220–0.234)	124.53 ± 5.80	124.73 ± 8.33	−1.335	0.193	−0.488 (−1.215–0.239)
2nd minute	124.40 ± 8.16	112.44 ± 7.78^a^^,^^b^	−2.734	0.011	−0.998 (−1.760–−0.236)	123.68 ± 5.40	107.31 ± 8.19^a^^,^^b^	−2.561	0.016	−0.935 (−1.692–−0.179)
3rd minute	124.80 ± 4.62	105.32 ± 4.89^a^^,^^b^	−3.704	<0.001	−1.353 (−2.151–−0.554)	124.60 ± 6.61	100.28 ± 7.40^a^^,^^b^	−2.862	0.008	−1.045 (−1.811–−0.279)
*F*	0.575	19.730				0.186	33.300			
*P*	0.633	<0.001				0.906	<0.001			
Lower-limb forward acceleration (G)										
10 s after start	0.92 ± 0.18	1.01 ± 0.20	1.141	0.264	0.417 (−0.307–1.141)	0.94 ± 0.35	1.01 ± 0.25	0.556	0.582	0.203 (−0.515–0.921)
1st minute	1.01 ± 0.30	0.95 ± 0.16	−0.634	0.532	−0.231 (−0.950–0.487)	1.07 ± 0.41	0.96 ± 0.23	−0.910	0.371	−0.332 (−1.053–0.389)
2nd minute	1.20 ± 0.25a	0.90 ± 0.32	−2.850	0.008	−1.041 (−1.807–−0.275)	1.23 ± 0.39	0.82 ± 0.26^b^	−3.372	0.002	−1.231 (−2.016–−0.446)
3rd minute	1.29 ± 0.25ab	0.87 ± 0.38	−3.616	0.001	−1.320 (−2.115–−0.526)	1.32 ± 0.38^a^	0.76 ± 0.24^a^^,^^b^	−4.747	<0.001	−1.733 (−2.581–−0.886)
*F*	6.993	0.722				2.909	3.414			
*P*	<0.001	0.543				0.042	0.023			
Foot clearance angle (°)										
10 s after start	20.36 ± 2.36	20.49 ± 2.00	0.163	0.872	0.059 (−0.656–0.775)	19.81 ± 1.67	19.66 ± 2.07	−0.226	0.823	−0.082 (−0.798–0.634)
1st minute	19.68 ± 2.58	19.90 ± 1.27	0.291	0.773	0.106 (−0.610–0.823)	19.46 ± 2.01	20.75 ± 3.62	1.201	0.24	0.439 (−0.286–1.163)
2nd minute	16.87 ± 2.61^a^^,^^b^	22.26 ± 2.95	5.303	<0.001	1.936 (1.059–2.813)	17.04 ± 2.39^a^^,^^b^	21.99 ± 2.22^a^	5.872	<0.001	2.144 (1.235–3.054)
3rd minute	15.23 ± 1.62^a^^,^^b^	22.54 ± 5.19	5.203	<0.001	1.900 (1.028–2.772)	15.60 ± 1.42^a^^,^^b^	24.48 ± 3.54^a^^,^^b^^,^^c^	9.016	<0.001	3.292 (2.172–4.413)
*F*	16.010	2.461				16.610	7.389			
*P*	<0.001	0.072				<0.001	<0.001			
Walking velocity (m/s)										
10 s after start	0.96 ± 0.17	0.93 ± 0.11	0.172	0.868	0.062 (−0.654–0.777)	0.98 ± 0.18	0.94 ± 0.09	0.802	0.431	0.293 (−0.426–1.013)
1st min	0.95 ± 0.11	0.92 ± 0.12	0.783	0.443	0.284 (−0.435–1.003)	0.95 ± 0.08	0.91 ± 0.10	1.213	0.238	0.441 (−0.283–1.165)
2nd min	0.97 ± 0.12	0.79 ± 0.08^a^^,^^b^	5.014	<0.001	1.830 (0.978–2.683)	0.97 ± 0.09	0.75 ± 0.09^a^^,^^b^	6.472	<0.001	2.361 (1.429–3.294)
3rd min	0.98 ± 0.11	0.70 ± 0.08^a^^,^^b^^,^^c^	7.932	<0.001	2.894 (1.870–3.918)	0.96 ± 0.10	0.67 ± 0.07^a^^,^^b^^,^^c^	9.251	<0.001	3.377 (2.263–4.492)
F	0.148	18.580				0.176	32.070			
P	0.866	<0.001				0.912	<0.001			

a indicates *P* < 0.05 compared to 10 s after start.

b indicates *P* < 0.05 compared to 1st minute.

c indicates *P* < 0.05 compared to 2nd minute.

**Table 3:** Comparison of energy expenditure between the SCP and control groups.

Energy consumption	Control (*n* = 15)	SCP (*n* = 15)	*t*	*P*	Cohen's d (95% CI)
10 s after start	0.104 ± 0.011	0.101 ± 0.010	1.853	0.075	0.675 (−0.063∼1.412)
1st minute	0.116 ± 0.011^a^	0.112 ± 0.010^a^	1.942	0.063	0.708 (−0.031∼1.448)
2nd minute	0.119 ± 0.012^a^	0.115 ± 0.012^a^	1.841	0.082	0.658 (−0.078∼1.394)
3rd minute	0.107 ± 0.012^b^	0.113 ± 0.012^a^	3.852	<0.001	1.404 (0.600∼2.209)
*F*	5.720	4.867			
*P*	0.002	0.005			

a indicates *P* < 0.05 compared to 10 s after start.

b indicates *P* < 0.05 compared to 1st minute.

**Table 4:** Comparison of RMS values between the SCP and control groups.

Parameter	Left					Right				
	Control (*n* = 15)	SCP (*n* = 15)	*t*	*P*	Cohen's d (95% CI)	Control (*n* = 15)	SCP (*n* = 15)	*t*	*P*	Cohen's d (95% CI)
Gastrocnemius muscle (%)										
10 s after start	1.00 ± 0.00	1.00 ± 0.00	—			1.00 ± 0.00	1.00 ± 0.00	—		
1st minute	1.26 ± 0.62	1.29 ± 0.59	−0.175	0.862	−0.064 (−0.780–0.652)	1.12 ± 0.42	1.10 ± 0.41	0.006	0.995	0.002 (−0.714–0.718)
2nd minute	1.41 ± 0.81^a^	0.85 ± 0.34^a^	−3.670	0.001	−1.340 (−2.137 to −0.543)	1.12 ± 0.48	0.77 ± 0.44^a^	−3.125	0.004	−1.141 (−1.917 to −0.365)
3rd minute	1.28 ± 0.60^b^	0.81 ± 0.45^a^	−3.173	0.004	−1.159 (−1.936 to ∼−0.381)	1.05 ± 0.32	0.59 ± 0.31^a^	−4.772	<0.001	−1.742 (−2.591 to −0.894)
*F*	7.970	0.739				0.144	6.576			
*P*	<0.001	0.029				0.866	0.003			
Tibialis anterior muscle (%)										
10 s after start	1.00 ± 0.00	1.00 ± 0.00	—			1.00 ± 0.00	1.00 ± 0.00	—		
1st minute	1.18 ± 0.37	1.33 ± 0.74	−0.344	0.733	−0.126 (−0.842–0.591)	1.15 ± 0.46	1.12 ± 0.40	−0.481	0.635	−0.176 (−0.893–0.542)
2nd minute	1.13 ± 0.25	0.87 ± 0.48^a^	−3.875	<0.001	−1.415 (−2.221 to −0.609)	1.14 ± 0.55	0.69 ± 0.24^a^	−3.595	0.001	−1.313 (−2.107 to −0.519)
3rd minute	1.16 ± 0.50	0.76 ± 0.42^a^	−4.563	<0.001	−1.666 (−2.504 to −0.828)	1.10 ± 0.43	0.72 ± 0.27^a^	−5.555	<0.001	−2.028 (−2.920 to −1.137)
*F*	0.023	4.311				0.045	8.928			
*P*	0.977	0.020				0.956	<0.001			

Values are mean ± standard deviation. Normalized RMS = RMS_t/RMS_10s. Independent-sample *t*-tests.

a indicates *P* < 0.05 compared to 1st minute.

b indicates *P* < 0.05 compared to 2nd minute.

**Table 5:** Parameter estimates of fixed effects from the linear mixed-effects model for gait parameters.

Parameter	Fixed effect	*β*	*S.E.*	*t*	*P*	*95% CI*
Single-step time	Time	−0.021	0.008	−2.481	0.013	−0.037 to −0.004
	Group	0.035	0.029	1.217	0.224	−0.021–0.091
	Side	0.011	0.022	0.497	0.619	−0.033–0.055
	Group × Time	0.005	0.012	0.421	0.674	−0.018–0.028
	Group × Side	−0.003	0.031	−0.095	0.924	−0.065–0.059
	Time × Side	−0.003	0.012	−0.278	0.781	−0.027–0.020
	Group Time × Side	0.020	0.017	1.167	0.243	−0.013–0.053
Stance phase	Time	−1.170	0.441	−2.655	0.008	−2.034 to −0.306
	Group	−0.263	1.400	−0.188	0.851	−3.007–2.482
	Side	1.390	1.166	1.192	0.233	−0.896–3.676
	Group × Time	1.803	0.623	2.892	0.004	0.581–3.024
	Group × Side	−0.620	1.649	−0.376	0.707	−3.853–2.612
	Time × Side	0.294	0.623	0.472	0.637	−0.928–1.516
	Group × Time × Side	1.085	0.882	1.231	0.218	−0.643–2.813
Swing phase	Time	1.170	0.441	2.655	0.008	0.306–2.034
	Group	0.263	1.4	0.188	0.851	−2.482–3.007
	Side	−1.390	1.166	−1.192	0.233	−3.676–0.896
	Group × Time	−1.803	0.623	−2.892	0.004	−3.024 to −0.581
	Group × Side	0.620	1.649	0.376	0.707	−2.612–3.853
	Time × Side	−0.294	0.623	−0.472	0.637	−1.516–0.928
	Group × Time × Side	−1.085	0.882	−1.231	0.218	−2.813–0.643
Gait cycle (s)	Time	−0.058	0.015	−3.787	<0.001	−0.088 to −0.028
	Group	0.017	0.049	0.345	0.73	−0.079–0.113
	Side	−0.019	0.041	−0.459	0.646	−0.098–0.061
	Group × Time	0.039	0.022	1.794	0.073	−0.004–0.081
	Group × Side	0.016	0.057	0.287	0.774	−0.096–0.129
	Time × Side	0.005	0.022	0.234	0.815	−0.037–0.048
	Group × Time × Side	−0.004	0.031	−0.115	0.908	−0.064–0.057
Step length	Time	0.001	0.004	0.142	0.887	−0.008–0.009
	Group	−0.005	0.014	−0.332	0.74	−0.033–0.023
	Side	0.009	0.011	0.811	0.417	−0.013–0.031
	Group × Time	−0.022	0.006	−3.692	<0.001	−0.034 to −0.010
	Group × Side	−0.007	0.016	−0.417	0.676	−0.038–0.024
	Time × Side	−0.004	0.006	−0.714	0.475	−0.016–0.007
	Group × Time × Side	0.003	0.008	0.363	0.717	−0.013–0.020
Cadence	Time	5.972	0.805	7.418	<0.001	4.394–7.550
	Group	−3.287	2.599	−1.265	0.206	−8.380–1.806
	Side	−0.829	2.13	−0.389	0.697	−5.003–3.346
	Group × Time	−2.139	1.139	−1.878	0.06	−4.370–0.093
	Group × Side	0.549	3.012	0.182	0.855	−5.355–6.453
	Time × Side	2.265	1.139	1.989	0.047	0.033–4.496
	Group × Time × Side	0.792	1.610	0.492	0.623	−2.363–3.948
Lower-limb forward acceleration	Time	0.128	0.024	5.402	<0.001	0.082–0.175
	Group	0.087	0.096	0.913	0.361	−0.100–0.275
	Side	0.034	0.063	0.547	0.585	−0.089–0.158
	Group × Time	−0.175	0.034	−5.212	<0.001	−0.241 to −0.109
	Group × Side	−0.019	0.089	−0.215	0.83	−0.194–0.155
	Time × Side	−0.002	0.034	−0.048	0.962	−0.068–0.064
	Group × Time × Side	−0.039	0.048	−0.816	0.415	−0.132–0.054
Walking velocity	Time	0.002	0.028	0.071	0.943	−0.053–0.057
	Group	−0.010	0.102	−0.098	0.922	−0.209–0.189
	Side	0.02	0.077	0.26	0.796	−0.131–0.171
	Group × Time	−0.095	0.042	−2.261	0.027	−0.177 to −0.012
	Group × Side	−0.030	0.109	−0.275	0.784	−0.243–0.183
	Time × Side	−0.012	0.042	−0.286	0.776	−0.094–0.070
	Group × Time × Side	0.003	0.06	0.05	0.96	−0.114–0.120
Foot clearance angle	Time	−1.820	0.281	−6.466	<0.001	−2.371 to −1.268
	Group	−0.744	0.816	−0.912	0.362	−2.343–0.855
	Side	−0.527	0.744	−0.708	0.479	−1.986–0.932
	Group × Time	2.670	0.398	6.710	<0.001	1.890–3.450
	Group × Side	−0.131	1.053	−0.125	0.901	−2.195–1.932
	Time × Side	0.314	0.398	0.788	0.431	−0.466–1.094
	Group × Time × Side	0.406	0.563	0.721	0.471	−0.697–1.509

Note. β, estimate; S.E., standard error; CI, confidence interval. Time coded as Time coded as 0 = 10 s after start, 1 = 1st minute, 2 = 2nd minute, 3 = 3rd minute; Group coded as 0 = Control, 1 = SCP; Side coded as 0 = Left, 1 = Right. The Group × Time interaction β represents the difference in temporal slope between the SCP and Control groups (units per minute). The Group × Time × Side interaction β represents the difference in temporal slope between the right and left limbs in the SCP group relative to the Control group.

**Table 6:** Parameter estimates of fixed effects from the linear mixed-effects model for energy expenditure.

Effect	*β*	*SE*	*t*	*P*	*95%CI*
Time	0.025	0.024	1.015	0.310	−0.023–0.072
Group	0.118	0.087	1.354	0.176	−0.053–0.288
Group × Time	0.068	0.034	1.988	0.047	0.001–0.135

Note. β, estimate; S.E., standard error; CI, confidence interval. Time coded as 0 = 10 s after start, 1 = 1st minute, 2 = 2nd minute, 3 = 3rd minute; Group coded as 0 = Control, 1 = SCP. The Group × Time interaction β represents the difference in temporal slope between the SCP and Control groups (units per minute). The Group × Time × Side interaction β represents the difference in temporal slope between the right and left limbs in the SCP group relative to the Control group.

**Table 7:** Parameter estimates of fixed effects from the linear mixed-effects model for RMS values.

Parameter	Effect	*β*	*SE*	*t*	*P*	*95%CI*
Gastrocnemius RMS (%)	Time	9.803	4.638	2.114	0.035	0.713–18.892
	Group	5.004	13.602	0.368	0.713	−21.656–31.664
	Side	−3.996	12.27	−0.326	0.745	−28.045–20.054
	Group × Time	−19.906	6.559	−3.035	0.002	−32.761–−7.051
	Group × Side	−0.175	17.352	−0.010	0.992	−34.186–33.836
	Time × Side	−8.123	6.559	−1.239	0.216	−20.978–4.732
	Group × Time × Side	2.811	9.275	0.303	0.762	−15.368–20.991
Tibialis anterior RMS (%)	Time	4.261	4.158	1.025	0.306	−3.890–12.411
	Group	11.527	12.241	0.942	0.346	−12.465–35.520
	Side	0.033	11.002	0.003	0.998	−21.530–21.597
	Group × Time	−16.104	5.881	−2.738	0.006	−27.631–−4.578
	Group × Side	−9.533	15.559	−0.613	0.54	−40.029–20.963
	Time × Side	−1.242	5.881	−0.211	0.833	−12.768–10.285

The sensors continuously transmitted angular velocity and acceleration data along three orthogonal axes (anteroposterior, mediolateral, and vertical) to the recorder, which automatically identified the activity type, analyzed the gait, and calculated duration, frequency, intensity, and energy expenditure. Data were processed using ActViewi software, IDEEA version 3.01.

**Figure 1:**
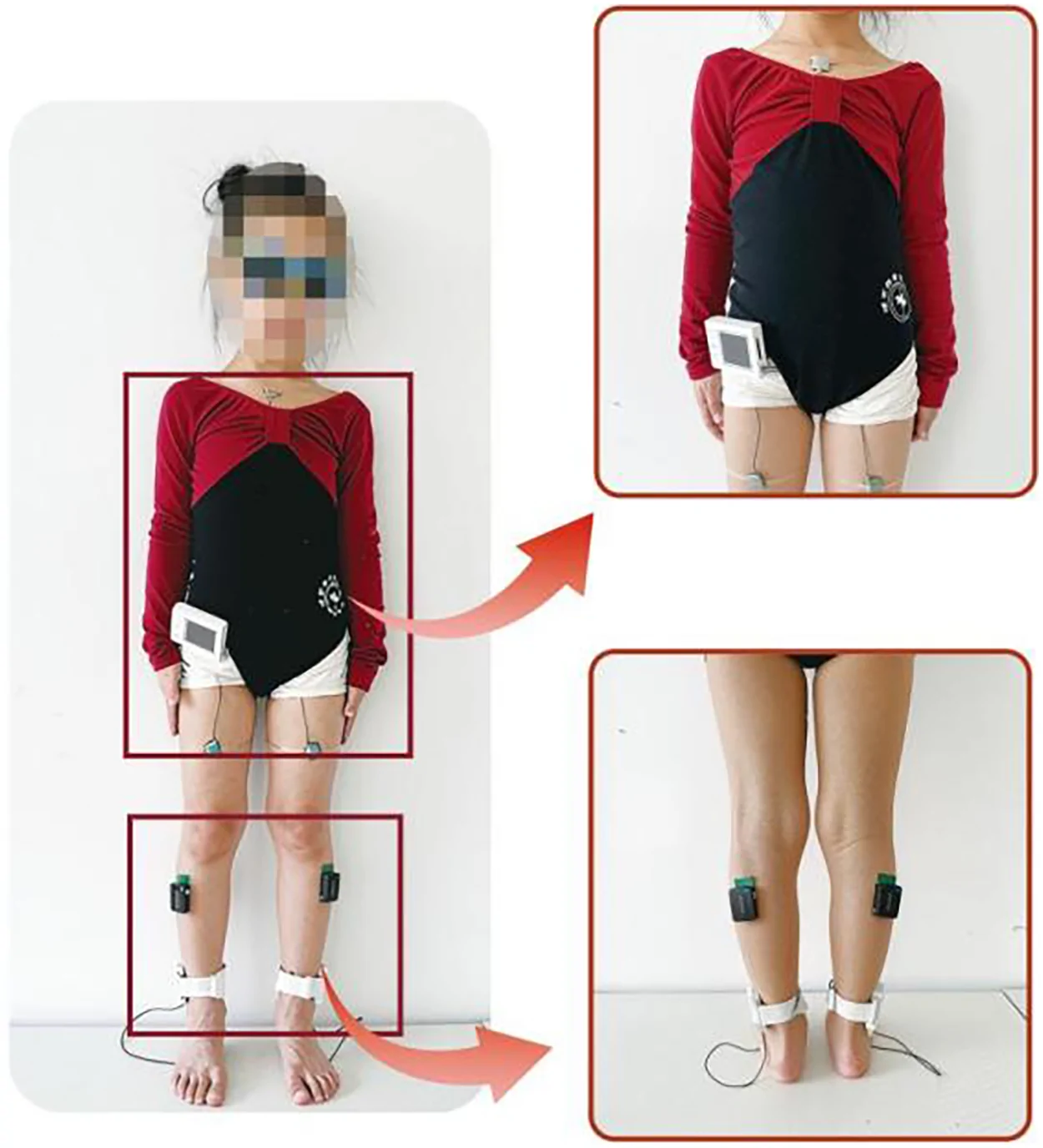
Gait and EMG electrode placement. Portable gait sensors were attached to the sternum, bilateral anterior thighs, and feet. Surface electromyography electrodes were placed over the bilateral tibialis anterior and lateral gastrocnemius muscles. All sensors were secured with medical adhesive tape.

Surface electromyography (sEMG) was recorded with a wireless system (Delsys, USA). Participants were barefoot, with lower legs fully exposed. sEMGwas recorded with a wireless system (Delsys Trigno, USA). Participants were barefoot, with lower legs fully exposed. Prior to electrode placement, the skin over each muscle belly was prepared by shaving excess hair, abrading with fine sandpaper, and cleansing with 70% isopropyl alcohol to reduce skin impedance to <5 kΩ. Bipolar Ag/AgCl surface electrodes (inter-electrode distance: 20 mm; electrode diameter: 10 mm) were positioned according to the SENIAM guidelines (15): the tibialis anterior electrodes were placed at one-third of the line between the fibular head and the medial malleolus, and the lateral gastrocnemius electrodes were placed at one-third of the line between the head of the fibula and the heel, over the most prominent muscle belly. Electrodes were secured with the system's built-in adhesive patches and additional hypoallergenic tape to minimize movement artifact. sEMG data were continuously collected at 2,000 Hz throughout the walking test (Figure 1).

### 2.4 Main outcome measures

#### 2.4.1 Gait and energy expenditure parameters

The following gait parameters were analyzed: single-step time, stance phase, swing phase, gait cycle duration, step length, cadence, forward acceleration of the lower limb, foot clearance angle, and energy expenditure. For each leg, cadence was defined as the reciprocal of the mean single-step time (steps per minute), allowing separate assessment of right- and left-limb rhythm. Among them, energy expenditure was derived from the IDEEA system's built-in algorithm, which integrates tri-axial acceleration data, cadence, walking duration, and body weight to estimate the metabolic energy cost. Energy expenditure was derived from the IDEEA system's built-in neural-network algorithm (ActViewi version 3.01), which integrates tri-axial acceleration data, cadence, walking duration, and body weight to estimate metabolic energy cost. To normalize for inter-individual differences in body mass, energy expenditure is expressed as kcal/kg/min. The IDEEA algorithm has been validated against indirect calorimetry in healthy adults and typically developing children during level walking, showing significant correlations and estimation accuracies exceeding 95% (16, 17). However, validation studies specifically in pediatric populations with hypertonic gait patterns remain limited (18). In children with cerebral palsy, the IDEEA has demonstrated lower reliability for temporal-spatial gait parameters and a tendency to overestimate step length while underestimating cadence compared to three-dimensional gait analysis (19).

#### 2.4.2 Surface electromyography parameters

Electromyographic activity of the bilateral gastrocnemius and tibialis anterior muscles was quantified using the root mean square (RMS) value, which reflects the average amplitude of muscle activity over time. Raw EMG signals were band-pass filtered (20–450 Hz), rectified, and smoothed using a 4th-order low-pass Butterworth filter at 10 Hz to generate a linear envelope consistent with standard gait EMG processing (20). The RMS amplitude was calculated over 250-ms epochs aligned to each gait cycle. Because maximal voluntary contraction (MVC) data could not be reliably obtained in young children with spastic cerebral palsy—a population known to have difficulty producing consistent maximal voluntary activation (21)—RMS values were normalized to the mean RMS amplitude recorded during the initial 10-second walking period for each participant, muscle, and side: Normalized RMS = (RMS_t/RMS_10s) × 100%. This approach expresses temporal changes in muscle activation as percentage change from the early steady-state walking phase, thereby preserving the trajectory of fatigue-related neuromuscular decline while reducing inter-individual variability in absolute amplitude (21). We acknowledge that peak dynamic normalization represents an alternative approach.

### 2.5 Statistical analysis

Data were analyzed with SPSS 26.0. Normally distributed continuous variables were expressed as mean ± standard deviation ($\bar{x}\pm s$). Between-group comparisons were conducted using independent-sample *t*-tests, while within-group comparisons were performed using paired-sample *t*-tests. Non-normally distributed data were expressed as median (P25–P75) and analyzed using the Mann–Whitney *U*-test for between-group comparisons and the Wilcoxon signed-rank test for within-group comparisons. Categorical data were expressed as proportions and compared using the chi-squared test. For comparisons between different time points within the same group, repeated-measures ANOVA was employed. Given the repeated-measures structure of the dataset (multiple time points and bilateral measurements nested within participants), linear mixed-effects models (LMMs) were employed as the primary analytical framework. For each gait and EMG parameter, a separate LMM was fitted with Group (SCP vs. Control), Time (10 s, 1st minute, 2nd minute, 3rd minute), and Side (Left vs. Right) as fixed factors, and Participant as a random intercept to account for within-subject correlations. The model initially included all two-way interactions (Group × Time, Group × Side, Time × Side) and the three-way interaction (Group × Time × Side). Significance of fixed effects and interactions was evaluated using likelihood ratio tests (LRT). Regression coefficients (β) and their 95% confidence intervals (CI) were extracted from the LMM fixed-effects estimates to quantify the magnitude and direction of group differences and interaction effects. Where significant Group × Time or Group × Time × Side interactions were detected, simple effects analyses were performed to examine (a) between-group differences at each time point and (b) within-group bilateral differences at each time point. All *post-hoc* pairwise comparisons were adjusted for multiple testing using the Holm–Bonferroni stepwise procedure to control the family-wise Type I error rate at 0.05. Statistical significance was set at *P* < 0.05 (two-sided). For energy expenditure, which was measured without side differentiation, an LMM with Group × Time fixed effects and Participant random intercept was fitted. EMG normalization: Because maximal voluntary contraction (MVC) data were not collected, raw RMS values of the gastrocnemius and tibialis anterior were normalized to the baseline (10-second) amplitude for each participant and side: Normalized RMS = (RMS_t/RMS_10s) × 100%. This approach expresses temporal changes in muscle activation as percentage change from the initial walking phase, thereby reducing inter-individual variability in absolute amplitude while preserving the temporal trajectory of muscle activation. The normalized RMS values were analyzed using the same LMM framework described above.

## 3 Results

### 3.1 Gait and energy expenditure analysis

#### 3.1.1 Bilateral gait parameters in the SCP group

**Figure 2:**
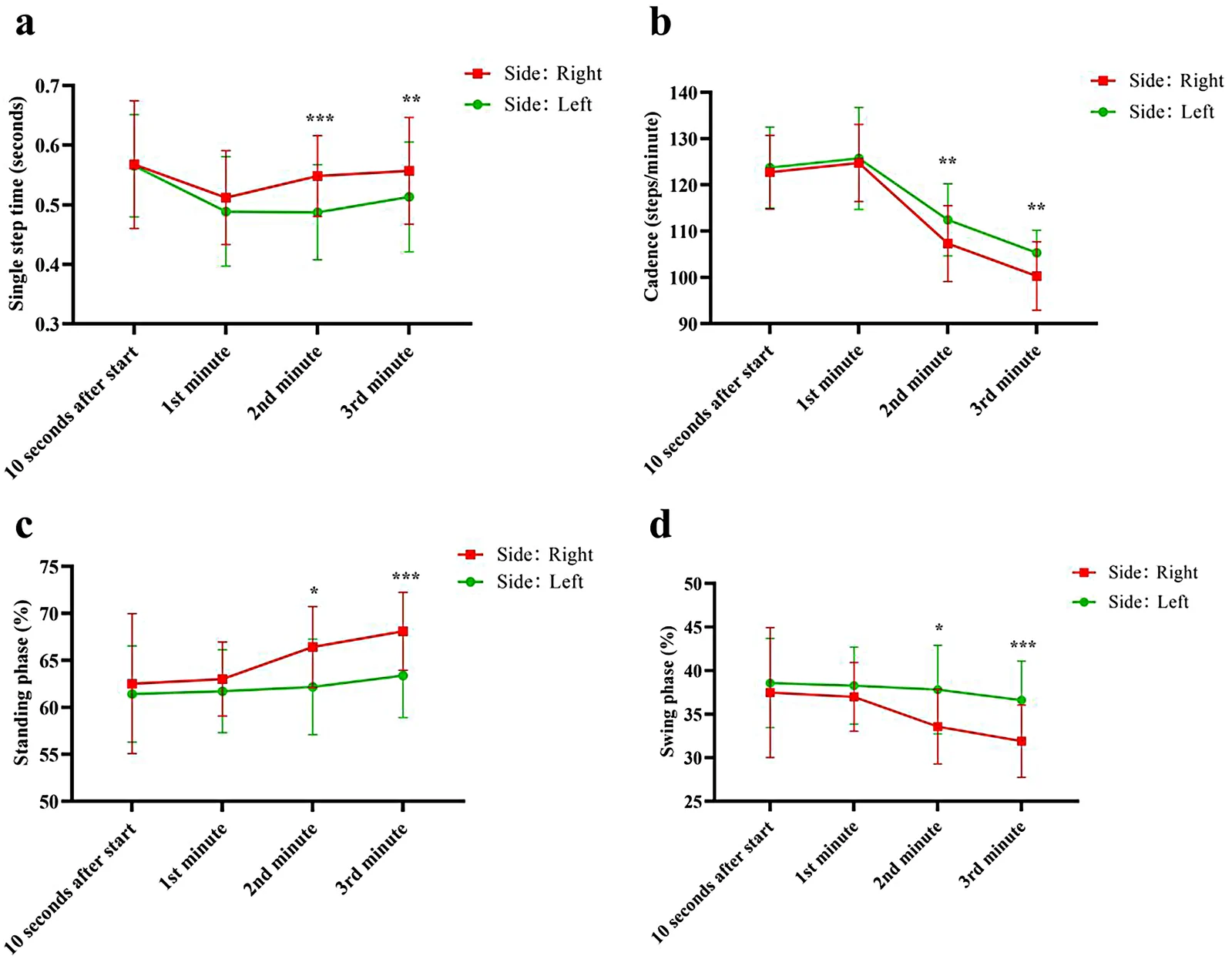
Comparison of left and right gait parameters in the SCP group. Changes in **(a)** single-step time, **(b)** cadence, **(c)** stance phase, and **(d)** swing phase across four time points (10 s after start, 1st, 2nd, and 3rd minute). Red lines represent the right limb and green lines represent the left limb. Data are presented as mean ± SD. Asterisks indicate significant left–right differences within the SCP group (*P < 0.05, **P < 0.01, ***P < 0.001).

This Figure 2 shows the dynamic changes in the gait parameters of the bilateral lower limbs of SCP children during continuous walking. As the walking time increases, the single-step time (Figure 2a), step frequency (Figure 2b), stance phase (Figure 2c), and swing phase (Figure 2d) all exhibit certain time-related changes. Compared to the initial stage of walking, at the 2nd and 3rd minutes, the single-step time on the right side increases, while the bilateral step frequency decreases; at the same time, the proportion of standing on the right side increases, and the proportion of swinging decreases. In contrast, the changes in the related parameters on the left side are relatively smaller. These results suggest that during continuous walking, some gait parameters of SCP children change over time, and some parameters show more significant changes on the right side (*P* < 0.05).

#### 3.1.2 Between-group comparisons of spatiotemporal gait parameters

Table 2 presents the between-group comparisons of spatiotemporal gait parameters for the left and right limbs separately. At gait initiation (10 s after start), no significant between-group differences were observed for any parameter on either side (all *P* > 0.05).

From the second minute onward, children with SCP exhibited significant alterations in multiple gait parameters compared with healthy controls. For the right limb, significant group differences emerged in single-step time at the 2nd and 3rd minutes, stance phase, swing phase, step length, cadence, lower-limb forward acceleration, foot clearance angle, and walking velocity (all *P* < 0.05).

For the left limb, significant group differences were also observed from the 2nd or 3rd minute in stance phase, swing phase, single-step time at the 3rd minute, gait cycle duration, step length, cadence, lower-limb forward acceleration, foot clearance angle, and walking velocity (all *P* < 0.05). Notably, effect sizes for right-sided parameters tended to be larger than those for the left side.

Within-group temporal trends are summarized by the repeated-measures F statistics in Table 2. In the control group, significant temporal changes were observed for stance phase, swing phase lower-limb forward acceleration, and foot clearance angle (all *P* < 0.05). In the SCP group, significant temporal changes included increased single-step time, decreased step length, decreased cadence, and decreased walking velocity (all *P* < 0.05).

#### 3.1.3 Linear mixed-effects model analysis of gait parameters

LMM analysis (Table 6) revealed significant Group × Time interactions for stance phase, swing phase, step length, lower-limb forward acceleration, foot clearance angle (*β* = 2.670, SE = 0.398, *P* < 0.001), and walking velocity (all *P* < 0.05), indicating divergent temporal trajectories between the SCP and control groups. The Group × Time interaction for cadence was no significant (*P* > 0.05). No significant Group × Time interactions were observed for single-step time or gait cycle duration (all *P* > 0.05).

Significant main effects of Time were observed for single-step time, stance phase, swing phase, gait cycle duration, cadence, lower-limb forward acceleration, and foot clearance angle (all *P* < 0.05). The main effect of Group was not significant for any parameter (all *P* > 0.05). All three-way interactions (Group × Time × Side) were non-significant (all *P* > 0.05), and the Group × Side interactions were also non-significant. A significant Time × Side interaction was observed for cadence (*P* = 0.047); all other Time × Side interactions were non-significant.

#### 3.1.4 Energy expenditure

Table 3 shows that both groups exhibited an initial increase in energy expenditure, peaking at the 2nd minute, followed by a decline. However, the SCP group maintained significantly higher energy expenditure than controls at the 3rd minute (*P* < 0.001). Within-group repeated-measures analysis indicated significant temporal changes in both groups (*P* < 0.05).

LMM analysis (Table 6) confirmed a significant Group × Time interaction for energy expenditure (*P* = 0.047), indicating that the temporal trajectory of energy cost differed between groups. Neither the main effect of Time (*P* = 0.310) nor the main effect of Group (*P* = 0.176) was statistically significant.

### 3.2 Surface electromyography analysis

Normalized RMS values (expressed as percentage change from the 10-second baseline) are presented in Table 4. At gait initiation, normalized RMS values were comparable between groups (all ∼100%). From the 2nd minute onward, the SCP group showed a bilateral decline in gastrocnemius and tibialis anterior activation relative to baseline. Between-group differences reached significance for the left gastrocnemius at the 2nd minute (*P* < 0.001) and 3rd minute (*P* < 0.001), and for the right gastrocnemius at the 2nd minute (*P* = 0.004) and 3rd minute (*P* < 0.001). For the tibialis anterior, significant between-group differences were observed bilaterally at the 2nd minute and 3rd minute (all *P* < 0.001).

Within-group temporal changes are reflected in the repeated-measures F statistics (Table 4). In the SCP group, significant temporal declines were observed in bilateral gastrocnemius and tibialis anterior (all *P* < 0.05). In the control group, significant temporal changes were limited to the left gastrocnemius (*P* < 0.001).

LMM analysis (Table 7) demonstrated significant Group × Time interactions for gastrocnemius RMS and tibialis anterior RMS (all *P* < 0.05), reflecting a more pronounced temporal decline in normalized EMG amplitude in the SCP group relative to controls. The main effects of Group and Side, as well as the Group × Side and Time × Side interactions, were not statistically significant (all *P* > 0.05). For gastrocnemius, the three-way interaction was non-significant (*P* = 0.762); the tibialis anterior model did not include this three-way interaction term.

## 4 Discussion

The present study employed a repeated-bout paradigm in which children with spastic cerebral palsy and healthy controls each completed six 3-minute overground walking trials with 2-minute seated rests. Linear mixed-effects model analysis revealed that children with SCP exhibited divergent temporal trajectories from healthy controls in stance phase, swing phase, step length, lower-limb forward acceleration, foot clearance angle, and walking velocity, with significant Group × Time interactions emerging from the second minute onward. Concurrently, bilateral gastrocnemius and tibialis anterior activation—quantified by normalized root mean square amplitude—showed a steeper temporal decline in the SCP group over the same interval. Energy expenditure, assessed without side differentiation, remained elevated in the SCP group into the third minute, whereas controls exhibited a declining metabolic trajectory suggestive of steady-state attainment. However, the interpretation of these temporal patterns requires careful consideration of several interrelated factors: the cumulative fatigue inherent to the repeated-trial design, the confounding influence of walking velocity on spatiotemporal kinematics, the distinct central and peripheral neurophysiological mechanisms that may underlie the observed EMG changes, the metabolic consequences of failed locomotor efficiency, and the methodological constraints of the study protocol.

We acknowledge that the repeated-trial design introduces a cumulative fatigue component that warrants careful interpretation. Each participant completed six 3-minute walking trials with only 2-minute seated rests between trials, yielding a total walking exposure of 18 min per session. In children with spastic cerebral palsy, peripheral neuromuscular fatigue—characterized by EMG median-frequency declines and altered muscle synergies—has been shown to emerge during prolonged walking protocols (22). A 2-minute rest interval may not be sufficient to achieve full metabolic recovery between walking bouts. Previous studies have demonstrated that restoration of high-energy phosphate metabolism after exercise requires several minutes and may be further delayed in populations with impaired muscle oxidative capacity. This consideration may be particularly relevant in children with spastic cerebral palsy, who exhibit increased susceptibility to lower-limb muscle fatigue during walking (23). Consequently, Trial 6 likely began with greater residual fatigue than Trial 1. To mitigate this confounder, descriptive statistics represent the within-participant mean across the six trials at each time point, and the primary inferential analyses were performed using linear mixed-effects models with Participant as a random intercept to account for within-subject correlations across trials and time points. We additionally tested a model that included Trial number (1–6) as a fixed effect; however, this term did not significantly improve model fit (likelihood ratio test, *P* > 0.05) and was omitted from the final parsimonious model. Therefore, the observed temporal changes from the second minute onward should be interpreted as gait deterioration occurring within the context of repeated walking bouts with brief recovery, rather than a single continuous 3-minute walk. This design enhances ecological validity for daily activity patterns—where children with SCP typically engage in intermittent rather than sustained walking—yet the generalizability of the “2-minute threshold” to a single continuous bout requires confirmation in future studies employing longer inter-trial rest intervals or continuous overground protocols.

The interpretation of temporal changes in stance and swing phase proportions must consider the confounding influence of walking velocity. Systematic reviews have demonstrated that gait speed exerts independent, moderate-to-large effects on spatiotemporal parameters: slower speeds are associated with prolonged stance duration and reduced cadence across pediatric, adult, and geriatric populations (24). In the present study, the LMM framework explicitly accounted for this by modeling Group × Time interactions while preserving the natural velocity decline observed in the SCP cohort. The significant Group × Time interactions for stance phase, swing phase, and cadence indicate that the temporal trajectories diverged between groups beyond what would be expected from velocity changes alone. Notably, the control group also exhibited minor velocity fluctuations across the 3-minute period, yet did not show the same directional shifts in stance–swing distribution or the magnitude of change observed in the SCP group. These findings suggest that the observed temporal parameter alterations in children with SCP reflect fatigue-related neuromuscular impairment rather than simple velocity-induced kinematic adjustments. Consistent with previous reports (25–27), children with SCP tended to exhibit prolonged single-step time, stance phase, and overall gait cycle duration relative to controls, with significant differences in selected parameters emerging from the second minute onward. The gait cycle comprises stance and swing phases, with the stance phase contributing to postural stability through coordinated limb and trunk adjustments (28). During typical development, stance phase duration decreases while swing phase duration increases, reflecting maturation of gait control (29). In children with SCP, swing phase duration is typically shortened and cadence reduced compared with healthy peers (30, 31), a tendency partially reproduced in the present cohort. Notably, swing phase duration in children with SCP was initially comparable to or slightly longer than that of controls during the first 10 s, but subsequently shortened, while cadence showed an initial increase followed by a gradual decrease.

The tibialis anterior and gastrocnemius muscles, primary contributors to ankle dorsiflexion and plantarflexion, are critical for maintaining gait stability and postural balance (32, 33). In this study, normalized RMS amplitudes for both muscles decreased over time, with a steeper decline in the SCP group from the second minute onward. The interpretation of this decline requires deeper neurophysiological context. In children with spastic diplegia, the gastrocnemius is often chronically hypertonic and mechanically shortened due to equinus deformity, leading to mechanical inefficiency and a predisposition to early exhaustion (34). The observed RMS decline could reflect either central fatigue—a failure of voluntary neural drive from the motor cortex or spinal motor neuron pool—or peripheral fatigue—failure at the neuromuscular junction or excitation–contraction coupling (35). Evidence from prior studies indicates that weakness in CP has a strong central component, with reduced motor-unit firing rates and impaired voluntary activation (36). However, the distal calf muscles in children with CP also exhibit greater fatigability during walking than thigh muscles, suggesting that peripheral mechanisms, including altered muscle fiber type composition and impaired metabolic efficiency, likely contribute. The bilateral nature of the RMS decline observed in our study, without significant side differences, is consistent with a systemic rather than focal fatigue process. The exact etiology in this specific cohort (whether predominantly central, peripheral, or mixed) cannot be determined from surface EMG alone and requires further investigation using techniques such as twitch interpolation or motor-unit decomposition.

Energy expenditure patterns further supported these observations. Children with SCP have consistently been shown to expend more energy than healthy peers both at rest and during ambulation (37). Excessive energy demands may exacerbate fatigue and contribute to gait instability (38). Our results extend these findings by suggesting that elevated energy expenditure in children with SCP may be associated with altered gait parameters and reduced neuromuscular activation. Although energy expenditure in controls peaked early and subsequently declined, children with SCP exhibited a more gradual reduction, with significantly elevated values persisting into the third minute. This sustained elevation indicates greater energetic demands to preserve gait stability and a reduced capacity to adapt efficiently to prolonged walking. It is worth noting that, due to the already high energy consumption during walking by the patient, and due to the synergistic contraction, abnormal synergy, and low efficiency of force transmission, the energy consumption further increases (39). When both gait parameters and muscle activation deteriorate simultaneously, the energy consumption continues to rise, indicating that the neuromuscular efficiency is gradually declining: as the activation degree of the ankle dorsiflexor and plantar flexor muscles decreases, the neuromuscular system is unable to effectively redistribute mechanical work, thereby causing an increased metabolic burden per unit distance (40). This study also indicates that this metabolic stress has already emerged within the first 3 min of continuous walking and will intensify with the increase in the number of walking sessions.

## 5 Limitations

Several methodological limitations should be acknowledged. First, the sample size (*n* = 15 per group) was determined according to established recommendations for pilot and feasibility studies, but remains modest for detecting subtle between-group differences after multiple-comparison correction; the findings should be considered hypothesis-generating and require confirmation in larger, multicenter cohorts. Second, spatiotemporal gait parameters were assessed using a wearable sensor system (IDEEA) rather than optical motion capture. While the IDEEA has demonstrated acceptable reliability for temporal–spatial parameters in children with cerebral palsy, its accuracy for kinematic variables such as foot clearance angle and joint angular excursions is inferior to three-dimensional gait analysis, and the system has been reported to overestimate step length while underestimating cadence in this population (9). Third, although turning phases were identified via gyroscope-derived directional-change detection and manually verified against video recordings, the exclusion of turning steps was based on algorithmic thresholds rather than force-plate–defined gait events; residual turning-related acceleration transients may have influenced energy expenditure estimates, particularly in children with SCP who exhibit wider turning radii and slower pivoting. Fourth, spasticity was graded using the Modified Ashworth Scale under static, passive conditions, which does not capture dynamic spasticity or velocity-dependent resistance during gait; the relationship between static MAS grades and functional muscle behavior during prolonged walking remains incompletely characterized. Finally, the 2-minute inter-trial rest was insufficient for complete fatigue recovery, and the repeated-trial design may have introduced order-related effects that cannot be fully disentangled from pure temporal gait deterioration.

## 6 Conclusion

In this exploratory study, children with spastic cerebral palsy demonstrated temporal alterations in spatiotemporal gait parameters, ankle muscle activation, and energy expenditure during repeated walking bouts. Group differences in single-step time, stance–swing distribution, foot clearance angle, and normalized gastrocnemius and tibialis anterior RMS amplitudes emerged from the second minute onward, while metabolic demands remained elevated in the SCP group. These findings indicate that sustained walking may impose greater neuromuscular and metabolic challenges in children with SCP. However, fatigue was inferred indirectly, fall risk was not directly assessed, and the repeated-trial design, small sample size, wearable sensor methodology, and static spasticity evaluation limit generalizability. Further studies using continuous walking protocols, validated fatigue measures, and direct functional outcomes are needed to clarify the clinical implications of these temporal changes.

## Data Availability

The original contributions presented in the study are included in the article/Supplementary Material, further inquiries can be directed to the corresponding author/s.
